# Innovations and the Use of Collimators in the Delivery of Pencil Beam Scanning Proton Therapy

**DOI:** 10.14338/IJPT-20-00039.1

**Published:** 2021-06-25

**Authors:** Daniel E. Hyer, Laura C. Bennett, Theodore J. Geoghegan, Martin Bues, Blake R. Smith

**Affiliations:** 1 Department of Radiation Oncology, University of Iowa, Iowa City, IA, USA; 2 Department of Radiation Oncology, Mayo Clinic Arizona, Phoenix, AZ, USA

**Keywords:** collimation, PBS, proton therapy

## Abstract

**Purpose:**

The development of collimating technologies has become a recent focus in pencil beam scanning (PBS) proton therapy to improve the target conformity and healthy tissue sparing through field-specific or energy-layer–specific collimation. Given the growing popularity of collimators for low-energy treatments, the purpose of this work was to summarize the recent literature that has focused on the efficacy of collimators for PBS and highlight the development of clinical and preclinical collimators.

**Materials and Methods:**

The collimators presented in this work were organized into 3 categories: per-field apertures, multileaf collimators (MLCs), and sliding-bar collimators. For each case, the system design and planning methodologies are summarized and intercompared from their existing literature. Energy-specific collimation is still a new paradigm in PBS and the 2 specific collimators tailored toward PBS are presented including the dynamic collimation system (DCS) and the Mevion Adaptive Aperture.

**Results:**

Collimation during PBS can improve the target conformity and associated healthy tissue and critical structure avoidance. Between energy-specific collimators and static apertures, static apertures have the poorest dose conformity owing to collimating only the largest projection of a target in the beam's eye view but still provide an improvement over uncollimated treatments. While an external collimator increases secondary neutron production, the benefit of collimating the primary beam appears to outweigh the risk. The greatest benefit has been observed for low- energy treatment sites.

**Conclusion:**

The consensus from current literature supports the use of external collimators in PBS under certain conditions, namely low-energy treatments or where the nominal spot size is large. While many recent studies paint a supportive picture, it is also important to understand the limitations of collimation in PBS that are specific to each collimator type. The emergence and paradigm of energy-specific collimation holds many promises for PBS proton therapy.

## Introduction

The field of proton therapy has grown rapidly since its initial proposal, fueled by the prospects of increased local tumor control and reduction of treatment toxicities in comparison to conventional photon-based treatment modalities. One of the most impactful developments since the advent of proton therapy has been pencil beam scanning (PBS), a treatment delivery modality where a narrow proton beam (beamlet) is steered within the treatment field by using a time-variant magnetic field [1, 2]. Pencil beam scanning offers 2 clinical advantages over passive scattering proton beam therapy: PBS can significantly spare healthy tissues proximal to the planning target volume (PTV) owing to its ability to create variable width spread-out Bragg peaks across the target; and with the addition of computer-aided beamlet weight optimization, PBS gives rise to intensity-modulated proton beam therapy (IMPT), which is analogous to IMRT in external photon beam therapy. From a technical standpoint, these advantages allow PBS to shape a dose distribution to closely match the target outline while minimizing the neutron production relative to its passive scattering counterpart [2–5]. For these reasons, the introduction of PBS was thought to largely mitigate the need for external apertures [1–3, 6]. However, both computational and clinical trial studies have recently emerged that paint an alternative picture: while proton therapy, in a theoretical sense, can provide superior healthy tissue sparing and increased target conformity over photon-based modalities, the conditions necessary to observe a significant clinical benefit are seldom achieved with PBS alone due in part to treatment planning uncertainties and increased lateral dose spread [7–10]. In any case, the potential benefit of proton beam therapy over other forms of external beam therapy may lie, depending on the clinical application, in a reduction of lateral beam penumbra, the reduction of integral dose, the increased radiobiological effectiveness (RBE) of protons, or in a combination of these factors. The clinical importance of these parameters is not particularly well understood, and the overall benefit of proton beam therapy is currently being tested in numerous clinical trials for various disease sites.

While protons exhibit a distinct depth-dose advantage over photon beams, a composite treatment plan's target conformity can be worse for the proton therapy treatment because of the lateral penumbra at the depth of the target [7]. For PBS without additional beam collimation, the size of the lateral penumbra is governed by the multiple Coulomb scattering in the patient and the initial size of the beamlet as it enters the patient. A recent treatment planning study by Widesott et al [11] suggests that for IMPT to have comparable organ-at-risk (OAR) sparing relative to advanced photon techniques necessitates that the size of a proton beamlet, defined by the standard deviation of the lateral spatial distribution in air, must be less than 3 mm, 4 mm, and 6 mm for prostate, head and neck, and malignant pleural mesothelioma cancers, respectively. Another investigation also demonstrated that a spot size of 4.3 mm at low energies is necessary for proton PBS to be superior to state-of-the-art photon therapies for intracranial radiosurgery [12]. As summarized in the Table, many of the worldwide, state-of-art facilities fail to meet these criteria.

**Table.** Beam spot widths for several proton therapy centers worldwide.

**Beamline facility**	**Width σ (mm)**	**MeV**
IBA DN system at the University of Pennsylvania [65]	4.7–2.6	100–225
IBA UN system at the University of Pennsylvania [66]	7.2–3.4	100–225
Hitachi system at the Mayo Clinic	5.3–2.0	71.3–228.7
Hitachi system at MD Anderson Cancer Center [67]	10–5	71.3–228.7
Centro Nazionale di Adroterapia Oncologica [68]	8.5–3.0	60–250
Mevion HYPERSCAN at Georgetown University Hospital [59]	15.7–4.1	28–227

Note: Beam widths are provided as in air sigma at isocenter.

The impact of spot size has also been shown to affect local tumor control between PBS and x-ray therapies [13, 14]. In each of these studies, spot size was a limiting factor toward a plan's performance relative to IMRT and tomotherapy treatments of head and neck targets. Thus, an underlying hypothesis behind the use of collimators in PBS is that collimated proton fields can provide superior healthy tissue sparing, compared to their uncollimated PBS counterparts, through the reduction of the lateral penumbra. This is especially true for lower beam energies that tend to have larger spot sizes and worsens for shallow targets that require external range shifters, such as for the head and neck.

**Figure 1.**
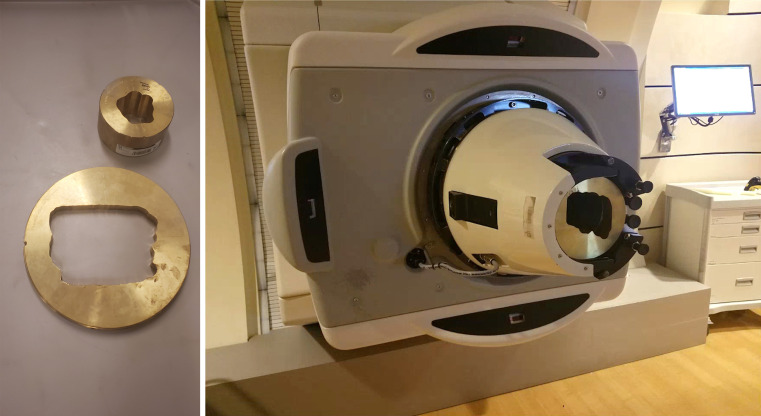
A brass aperture milled for small and large field (left) and a brass aperture mounted in the IBA Universal Nozzle for pencil beam scanning at the Northwestern Proton Center (right). Image courtesy of Dr Mark Pankuch, Northwestern Chicago Proton Center.

**Figure 2.**
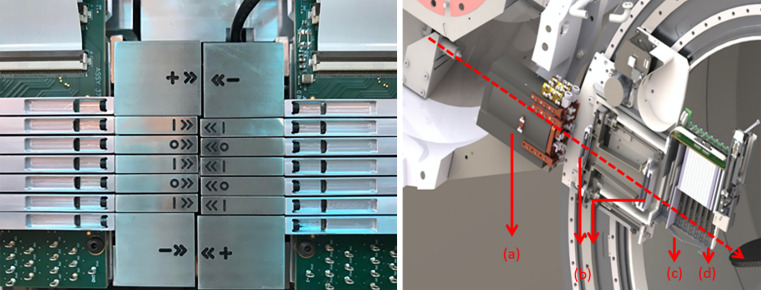
(Left) Frontal view of the Adaptive Aperture while the leaves are fully closed. Image taken by and courtesy of Kang and Pang [59]. (Right) The HYPERSCAN treatment head consisting of the (a) scanning magnet, (b) ionization monitor chambers, (c) variable range shifter (energy selector), and (d) the adaptive aperture.

**Figure 3.**
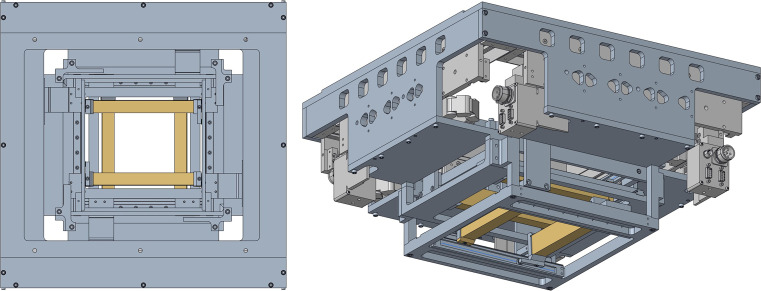
(Left) Beam's-eye-view of the DCS with the set of 4 orthogonal trimmer blades (shown in yellow) retracted from the center of the field. (Right) The side view of the whole DCS as it would be mounted on a PBS nozzle. Abbreviations: DCS, dynamic collimation system; PBS, pencil beam scanning.

For many facilities, external collimators may be necessary to increase target conformity or assist in OAR avoidance, since the spot sizes are an inherent characteristic of the beam line and accelerator. In response to this limitation, a multitude of existing collimating techniques have been adapted from passive scattering proton therapy and contemporary photon IMRT. Fixed, per-field apertures, as shown in Figure 1, have been used in the treatment of intracranial targets near critical structures [15–17] and to spare the lens, macula, and optic disc during intraocular treatments [18]. Multileaf collimators have also been adapted from x-ray Linacs [19, 20]. However, a large effort has been made within the last few years to develop novel collimating technologies tailored toward PBS [21–23]. The dynamic collimation system (DCS), as shown in Figure 3, was initially proposed by Hyer et al [24] in 2013 as a sliding-bar collimator capable of energy-layer collimation in spot scanning proton therapy that can sculpt the dose distribution uniquely to each planned energy layer. In addition to the work that has been published developing this technology, the DCS has also served as a vehicle to develop several treatment-planning–specific optimization methods and perform proof-of-principle treatment planning studies showcasing the capabilities of energy-layer–specific collimation in PBS. Following the DCS, the Adaptive Aperture by Mevion Medical Systems (Littleton, Massachusetts), as shown in Figure 2, is a multileaf collimator (MLC)–based energy-specific collimator that is actively used across a wide range of PBS proton therapy treatments [25, 26].

Within the past decade, the development and application of external collimating devices in PBS has played an increasingly popular role in improving PBS proton therapy, particularly with intracranial and head and neck cancers. To date, the most common collimators discussed in the literature for use with PBS include patient-specific apertures, MLCs/Mevion Adaptive Aperture, and the DCS. Given the variety of collimating devices currently available, it is important to understand their inherent differences and how these differences may manifest clinically with respect to both their technical design advantages and disadvantages as well as the treatment planning methods and optimization techniques that are unique to each device. Within this report, collimating technologies are referred to by their manufacturer and advertised product name for identification and comparison purposes only. The authors do not endorse any of these commercial or research collimating devices or recommend that any of these collimating devices are necessarily the best available for any proton therapy treatment.

## Materials and Methods

There are universal features consistent among all external collimating devices that influence their performance and efficacy. These include the choice of collimating materials, inclusion and positioning of an external range shifter, relative positioning to the patient, and technology- specific algorithmic approaches that have been developed for clinical implementation. Active research in collimated PBS has used a mixture of Monte Carlo methods and clinical dosimetry to design and develop apertures, multileaf collimators, and sliding-bar collimators in addition to treatment planning studies that have evaluated their efficacy [15, 16, 21–23, 27–32]. Quality assurance of collimators and collimated treatment plans consist of measurements prioritizing field-edge penumbra and in-phantom, planar dose distribution agreement among multiple water-equivalent depths and have been done using film dosimetry, micro-ionization chambers, ion chamber arrays, and scintillation plates [25, 33–36].

### Apertures

Brass apertures, in particular, have been used to collimate treatment fields since the first patient was treated with passive scattering in the United States [37]. In addition to its machinability, brass is a cost-effective metal that is ideal for aperture construction given that apertures must be created uniquely for each treatment field. By design, an aperture will generally collimate the largest projection of a target in the beam's eye view, making the beamlet-to-aperture margin an important parameter, as the lateral projection will affect various energies differently depending on the tumor shape and surrounding tissues. A notable exception is in the use of an aperture for patch combos, in which a portion of the target is occluded to prioritize OAR sparing.

Historically for passive scattering systems, the dimensions of the aperture are often defined as an expansion about the PTV in the beam's eye view [38]. While there is no standard method for determining aperture margins in PBS, similar methods have been adapted from passive scattering practices to determine the physical dimension and spot positions for aperture-based treatments [16, 39]. Retrospective methods have also been used with advanced beamlet models for novel technologies, such as proposed by Wang et al [40], to select an aperture margin from the maximum centroid spot shift.

Treatment planning studies have investigated the use of patient-specific apertures for single field, uniform dose (SFUD) treatment plans [15, 16, 41] and IMPT-optimized plans [39, 42]. SFUD optimization was a common choice for studies using Monte Carlo simulations among traditional Monte Carlo algorithms, such as Geant4 [41], as well as the fast Monte Carlo algorithms available in commercial treatment planning systems, such as RayStation (RaySearch Laboratories AB, Stockholm, Sweden) [16, 42].

### Multileaf Collimators

As with apertures, MLC-based collimation systems in proton therapy have been used for shallow targets situated near radiosensitive OARs, such as those in the head and neck region [43, 44]. Early PBS MLC systems adopted tungsten leaves from their photon counterparts but have since been customized with advantageous metals and mechanical designs resulting from a mixture of Monte Carlo and clinical dosimetry studies [21, 22, 30, 44] and PBS-specific optimization methods [45]. Owing to their large mechanical footprint and mass, MLCs must be installed in the gantry head. As a result, this tends to increase the collimator-to-patient air gap, resulting in a larger penumbra in practice [16, 20].

Treatment planning studies have been conducted by using Monte Carlo [44, 46] and RayStation [43] to determine the advantages between MLC-based PBS in comparison with standard PBS both with SFUD and IMPT techniques. At least 1 study has simulated the treatment of lung tumors in small animal irradiation by using the Mevion Adaptive Aperture, which consists of nickel collimating leaves placed downstream of a Lexan polycarbonate energy modulation system [26, 46]. The viability of MLC-based PBS combined with contour scanning as well as grid scanning has also been explored [44] in addition to studying the limits of collimation during PBS, which includes interleaf transmission, scalloping effects from large leaf sizes, and beam energy constraints [21, 45].

### DCS/Sliding-Bar Collimators

Conceptually, the DCS is a sliding-bar collimator that uses a common set of 3-cm-thick orthogonal trimmer blades placed downstream of an external 4-cm-thick polyethylene range shifter to intercept the proton beam as it scans near the target periphery [24]. To provide focused collimation, the DCS rotates the collimators to match the beam deflection angle as the collimators travel in a single plane, which can improve the lateral conformity [47, 48]. By design, the DCS can effectively provide any aperture shape for all energy layers. Like an aperture, the DCS is a nozzle-mounted accessory, which allows the device to be placed near the patient and integrated among a variety of beamlines. Unlike apertures or MLCs, the conceptual framework and design of treatment planning optimizers specific to the DCS has treated collimation on a per-beamlet basis, rather than as a holistic fluence that is collimated by a common projection. This is apparent when considering how PBS systems can scan faster that the translation speed of the collimator, introducing treatment time penalties. Spot and collimator placement algorithms have been developed to minimize collimator travel and treatment times while maintaining coverage [49, 50].

The ability of the DCS to generate conformal treatment plans has been evaluated on both brain and head and neck cases within an in-house treatment planning system [51–53] using both SFUD and IMPT optimization algorithms [32]. Given the high conformity offered by the DCS, the robustness of the device to setup error and random uncertainty in beam spot and collimator rod position is of concern owing to the potential for any error to change the overall dose distribution. A mixture of Monte Carlo methods and model-based beamlet algorithms within a research treatment planning system has been used to investigate the robustness of the trimmer positioning by evaluating the treatment variability from randomly distributed uncertainties in spot and trimmer position and DCS mounting alignment [54].

## Results

From a collimator material selection standpoint, nickel provides the best combination of small scattering angle with low neutron production and activation when compared to alternatives such as brass, tungsten, iron and plastic [28, 31, 32, 55]. While plastic produces the least number of neutrons, it requires the largest thickness of material, which in turn leads to larger air gaps and subsequently larger spot sizes [27]. Tungsten performs well to collimate protons, but neutron activation and production are worse than for nickel [27, 30]. There is some benefit to using brass compared to nickel or tungsten for apertures as it is cheaper, easier to machine, and does not produce toxic dust during the manufacturing process [27, 56].

A range shifter is often necessary to cover superficial targets but degrades lateral conformity owing to scattering of the beam, therefore the placement and material selection of the range shifter should also be considered when designing a collimation system. When considering the placement of the range shifter, the material and thickness of both the range shifter and collimator dictate the best sequence of the two devices to minimize lateral penumbra. This means the optimal sequence will be specific to individual collimation systems [15, 23, 29]. In general, for most collimation systems used in PBS proton therapy, the range shifter should be placed upstream of the collimator [29].

For all collimation systems, the distance from the patient to collimator should be minimized to reduce the geometric growth of the lateral penumbra [16]. This is best accomplished by designing a collimator that has a small footprint to allow for clearance around the patient anatomy. Both apertures and the DCS have a smaller footprint than MLC-based designs and should provide minimal air gaps when combined with a telescoping nozzle.

### Apertures

The selection of an aperture's blocking margin, which defines the aperture shape, is an important parameter to consider. One strategy chooses the aperture margin from the maximum shift of the beamlet center owing to collimation [40]. Practically speaking, most studies have used aperture margins of 4.0 to 15.0 mm [17, 39, 40, 57, 58]. The second consideration is whether to mill the aperture to match the divergence of the beam. By using divergent-cut apertures, the dose near the field edges at the surface can be reduced up to 9.5% and neutron contamination can be reduced by 6% at the surface [48].

Patient-specific apertures have been shown to reduce the mean dose to healthy organs [17, 39] and offer superior dose falloff [15, 16] when compared with uncollimated PBS. The improvement in the plan quality offered by patient-specific apertures is dependent on several factors, which include spot size, air gap, and the depth of the target in tissue. Larger spot sizes (8 mm and beyond) benefit the most from the addition of apertures [17] followed by mid-sized spots (∼5 mm σ) [12]. Air gaps less than 5 cm were associated with greater improvement in the penumbral width and target conformity when an aperture was introduced into a PBS field for shallower targets [16]. When considering complicated targets, such as those found in the head and neck, apertures may not be the optimal collimator choice, since they cannot discriminate collimation among different energy layers, but may still afford sparing of specific OARs [17].

### Multileaf Collimators

It has been shown that a theoretical unfocused, nickel MLC can reduce the lateral penumbra from 1.3 mm to 0.3 mm at 72 MeV. At 118 MeV, the lateral penumbra was reduced from 0.9 mm to 0.5 mm. At energies higher than 159 MeV, there was no benefit for the MLC owing to multiple Coulomb scattering in the patient becoming the dominant source of penumbra size [21]. The Mevion Adaptive Aperture has also been shown to provide penumbra reduction over a range of energies and is capable of per-spot collimation to improve in-field gradients. The initial spot size of the Mevion system is much larger than a traditional PBS delivery system and therefore the results of the penumbra reduction are much more drastic than seen with other systems. For example, between 2-mm and 13-mm reduction in the field penumbra width of a 10 cm × 10 cm uniformly scanned field has been observed between beam energies of 227 MeV and 28 MeV, respectively, where a 28-MeV spot has a nominal spot size σ of 26 mm at isocenter [59].

MLC-based PBS treatment planning studies have been observed to reduce dose to healthy tissue outside of the PTV [43, 44]. A study by Sugiyama et al [43], examining patients with maxillary sinus cancer undergoing IMPT with and without MLC, found that plans developed with MLCs were able to reduce the maximum dose to the optic nerve and the mean dose to the ipsilateral nerve when compared with the non-MLC plans [43]. As MLCs have limited benefit at greater depths, the combination of collimated PBS and contour scanning to treat targets at depths less than 15 cm water equivalent thickness creates the greatest improvement in healthy tissue sparing when compared with plans generated with contour-scanned PBS alone; however, target dose homogeneity was not shown to be significantly different between the 2 cases [44].

### DCS/Sliding-Bar Collimators

The DCS has been the subject of several recent investigations about collimator design and potential use cases. From a design perspective, the placement of the collimators downstream of the range shifter reduces the lateral spread by 15% to 30%, compared to placing the collimators upstream of the range shifter [29, 47]. Polyethylene was chosen as the range shifter material because it has the lowest scattering angle of all suitable plastics [60]. Through the use of novel sequencing and grouping algorithms, it has been shown that the time penalty from using the DCS was on average 60 seconds per treatment field while maintaining high levels of conformity [49]. The robustness of DCS plans has also been evaluated. As with all collimators, the dose distribution of an individual beamlet is highly sensitive to the relative distance of the centroid of the beamlet to the medial edge of the collimator. To combat this sensitivity, a minimum allowable off-axis trimmer distance was implemented for the DCS to improve robustness. For treatments planned by using a model of the Northwestern IBA Universal Nozzle (UN) beamline, introducing a small 1.5-mm offset from the center of the beamlet to the face of the trimmer improved the PTV D95% robustness to within 2% of the nominal coverage even when accounting for uncertainties from the mounting alignment, spot and trimmer positioning [54].

Head and neck PBS plans generated by using the DCS have consistently demonstrated greater reduction in mean dose to healthy tissue adjacent to the target and improved target conformity relative to uncollimated PBS plans as well as fixed aperture plans [51, 52, 61]. When comparing the DCS to uncollimated treatment to both the IBA Universal and Dedicated Nozzle systems, the DCS reduced the mean dose delivered to the nearby 10-mm margin of healthy tissue surrounding the target by 11.5 % to 13.7% for brain treatments and 6.3% to 7.1 % for head and neck targets in comparison to uncollimated treatments with an equivalent prescribed dose to the 95% PTV volume [51]. Significant OAR avoidance could also be achieved by using the DCS relative to an uncollimated treatment with an equivalent PTV coverage, as much as an 86% reduction to the left optic nerve during a dual-field chordoma treatment and a 35% reduction to the spinal cord during a treatment of a squamous cell carcinoma of the left supraglottic larynx [51]. Compared to uniform scanning plans generated with a brass aperture, DCS-generated plans were subject to less secondary neutron contamination owing to the use of nickel as a preabsorber material; however, they still present an increase in neutron generation as compared with an uncollimated PBS plan [31].

## Discussion/Conclusion

External collimating devices play an important role in low-energy PBS proton therapy and will most likely become a common accessory for PBS systems with a nominally large spot size, especially at lower beam energies that include an external range shifter where spot sizes may prohibit a notable dosimetric advantage in healthy tissue sparing or OAR avoidance when compared to contemporary photon treatments. While proton therapy delivery technology continues to improve and spot sizes continue to get smaller, these small spots open the door to increased sensitivity of spot misplacement [62]. In this regard, collimating a larger spot size can improve the achievable conformity near to that of a smaller spot size [63] with the added benefit of improving target coverage robustness, which can be a concern for moving targets or intrafraction motion [64].

Commercial availability remains an important factor to consider when implementing new technologies into the treatment planning and delivery workflow. Apertures remain the most commercially available and can either be machined in-house or ordered from a supplier. Although their performance does not match that of the energy-layer–specific systems, they are much cheaper and generally do not require any specialized software for treatment planning given that most contemporary PBS proton therapy treatment planning systems are capable of modeling single, per-field apertures. When an external range shifter is necessary to cover depths proximal to the minimum energy of the beamline, the sequence of collimator and range shifter can influence the beam spread. For the DCS and Adaptive Aperture, both elect to place the collimator downstream of the range shifter. The DCS and Adaptive Aperture require extensively more hardware and software to operate than conventional apertures. Each system requires a special mount that is beamline specific. Additionally, only a few commercial treatment planning systems are capable of treatment planning with energy-layer–specific collimation, namely Astroid from .decimal and RayStation for the DCS and Adaptive Aperture, respectively. While a clinical prototype exists for the DCS, the Adaptive Aperture is currently the only commercially available energy-layer–specific collimator.

A reduction in spot size from collimation during PBS treatment plans is associated with a reduction in dose to healthy tissue and steeper dose falloff for shallow targets. Beyond a depth of 15 to 20 cm, apertures, MLCs, and the DCS rarely provide a notable benefit in reducing the penumbra owing to the increased multiple Coulomb scattering relative to the nominal spot penumbra size [16, 21, 44].

However, for instances where the initial spot penumbra is very large, collimation may still provide some benefit for higher nominal beam energies as a field-defining collimator, which has been demonstrated with the Mevion Adaptive Aperture system [59]. While there is a clear benefit from improvements to the primary beam, collimation induces unwanted secondary radiation from nuclear interactions between the collimating material and the incident proton beam. As such, the use of collimation in PBS carries an increased risk of secondary complications due to increased neutron dose as compared with uncollimated PBS proton therapy [32]; however, the risk is lower than what is expected for uniform scanning with brass apertures. Additionally, the relative risk due to increased neutron dose may be mitigated by the improved conformity provided by these technologies. A study by Geng et al [58] found the lifetime attributable secondary malignancy risk for in-field tissues treated with a patient-specific aperture to be reduced for both large and small spot sizes when compared with standard PBS alone. The DCS was likewise observed to have a reduction in acute side effects such as brain necrosis among intracranial treatment plans [32].
